# Supplementary material for: Ceramsite production from sediment in Beian River: characterization and parameter optimization
Source: R Soc Open Sci. 2019 Aug 14;6(8):190197. doi: 10.1098/rsos.190197 (PMC6731694; doi:10.1098/rsos.190197)
Supplement: Experimental results of determination of polycyclic aromatic hydrocarbons [file rsos190197supp1.docx]

Table S1 Experimental results of determination of polycyclic aromatic hydrocarbons

| Upstream | | Midstream | | Downstream | |
| --- | --- | --- | --- | --- | --- |
| Dry season | Wet season | Dry season | Wet season | Dry season | Wet season |
| Stigmasterol, 22,23-dihydro- | Cholest-5-en-3-ol (3.beta.)- | Cholest-5-en-3-ol (3.beta.)- | Levo-5.beta.-dihydronorgestrel-methyloxime-TMS | Cholest-5-en-3-ol (3.beta.)- | Cholest-5-en-3-ol (3.beta.)- |
| Cholestane,3-ethoxy-, (3.beta.,5.alpha.)- | Cholestanol | Cholestanol | Cholest-5-en-3-ol (3.beta.)- | Cholestanol | Cholest-3-ene, (5.alpha.)- |
| Cholest-5-en-3-ol (3.beta.)- | 1,2-Benzenedicarboxylic acid, diisooctyl ester | Octadecane | *3-Methyl-1-phenyl-2-azafluorene* | Di-n-octyl phthalate | Cholest-3-ene, (5.alpha.)- |
| Cholest-3-ene, (5.alpha.)- | Octadecane | Octadecane | *Anthracene, 9,10-dihydro-9,9,10-trimethyl-* | *Triphenylene* | Heptacosane |
| 19-Norcholesta-1,3,5(10)-trien-6-one | Eicosane | Di-n-octyl phthalate | 1,2-Benzenedicarboxylic acid, mono(2-ethylhexyl) ester | *Benz[a]anthracene* | Di-n-octyl phthalate |
| Octacosane | *Pyrene* | Eicosane | Eicosane | *Benzo[ghi]fluoranthene* | Eicosane |
| Ergost-14-ene, (5.alpha.)- | *Fluoranthene* | Tetracosane | *Pyrene* | 9-Octadecenamide, (Z)- | Tetracosane |
| 1,2-Benzenedicarboxylic acid, mono(2-ethylhexyl) ester | *Fluoranthene* | 5-Octadecene, (E) | Cyclohexadecane | 1-Nonadecene | Eicosane |
| Tetradecanamide | *Phenanthrene* | 1,13-Tetradecadiene | *Fluoranthene* | *Pyrene* | 2(3H)-Furanone, 5-dodecyldihydro- |
| *Pyrene* | Heptadecane | 1-Nonadecene |  | 5-Octadecene, (E)- | 1-Octadecene |
| 1-Hexadecene |  | Cyclohexadecane |  | *Fluoranthene* | *Fluoranthene* |
| *Pyrene* |  |  |  | *Fluoranthene* | 2-Heptadecanone |
| *Fluoranthene* |  |  |  | *Dibenzothiophene* | 7-Hexadecene, (Z)- |
| *Fluoranthene* |  |  |  | *9H-Fluoren-9-one* | Octadecane |
| Cyclic octaatomic sulfur |  |  |  | Cyclohexadecane | Cyclohexadecane |
| Cyclic octaatomic sulfur |  |  |  |  |  |
| Cyclic octaatomic sulfur |  |  |  |  |  |
| *Phenanthrene* |  |  |  |  |  |
| *Phenanthrene* |  |  |  |  |  |
