# Supplementary material for: Ceramsite production from sediment in Beian River: characterization and parameter optimization
Source: R Soc Open Sci. 2019 Aug 14;6(8):190197. doi: 10.1098/rsos.190197 (PMC6731694; doi:10.1098/rsos.190197)
Supplement: Ceramsite performance test and comparison table [file rsos190197supp2.docx]

Table S2 Ceramsite performance test and comparison table

| Items | Sediment Ceramsite | index |
| --- | --- | --- |
| The sum of the breaking rate and the wear rate (*C_b_*/%) | 4.7 | ≤6 |
| Mud content (*C_s_*/%) | 0.56 | ≤1 |
| Hydrochloric acid soluble rate (*C_ha_*/%) | 1.2 | ≤2 |
| Specific surface area (*S_w_*/cm^2^/g) | 2.95×10^4^ | ≥0.5×10^4^ |
| Water absorption (%) | 3 | ≤10 |
| Bulk density (kg/m^3^) | 620 | 610-700 |
| Pressure strength (Mpa) | ≥5 | ≥3 |

* All the data were based on dry materials
